# Enhancement Strategies of Calcium Looping Technology and CaO‐Based Sorbents for Carbon Capture

**DOI:** 10.1002/smll.202412463

**Published:** 2025-02-28

**Authors:** Zirui Wang, Chenyang Ma, Alexander Harrison, Khulud Alsouleman, Mingchen Gao, Zi Huang, Qicheng Chen, Binjian Nie

**Affiliations:** ^1^ Department of Engineering Science University of Oxford Oxford OX1 3PJ UK; ^2^ Department of Computer Science University of Oxford Oxford OX1 3QG UK; ^3^ Energy Process Engineering and Conversion Technologies for Renewable Energies Technische Universität Berlin 13353 Berlin Germany; ^4^ School of Energy and Power Engineering Northeast Electric Power University Jilin 132012 China

**Keywords:** calcium looping, CaO‐based sorbents, carbon capture, machine learning

## Abstract

As global warming intensifies and energy resources deplete, carbon capture and sustainable energy conversion technologies gain increasing importance. Among these, calcium looping (CaL) technology has demonstrated promising cost‐effectiveness and ease of integration with other systems. However, severe sintering of CaO‐based sorbents occurs during cyclic carbonation and calcination, resulting in a significant decrease in CO_2_ capture capacity and stability. This paper reviews enhancement strategies in aggregate for synthetic CaO‐based sorbents over the past 10 years, compiling a tabular dataset of 1042 reported materials, to compare the effects of synthesis methods and operation conditions on decay rate and CO_2_ capture capacity. Sol‐gel, combustion, and template synthesis methods are recommended for producing high porosity CaO‐based sorbents. The calcium precursors and organic acids used during synthesis, and addition of dopants, also play important roles in affecting the sorbent performance. This paper also examines the relationship between material synthesis, operation conditions, and performance of CaO‐based sorbents to determine the feasibility of applying machine learning technology in materials development. This paper also discusses several possible artificial intelligence strategies with potential for designing innovative CaO‐based sorbents suitable for long‐term industrial applications, with the XGBoost model providing promising predictive capacity, particularly when working with relatively small, tabular, datasets.

## Graphical abstract

A comprehensive review of Calcium Looping (CaL) technology for CO_2_ capture is presented, compiling a tabular dataset of 1042 reported materials to compare the effects of synthesis methods and operation conditions on decay rate and CO_2_ capture capacity. Several machine learning strategies are considered for facilitating CaL process design and process optimizations, accelerating materials development and sustainable carbon capture.

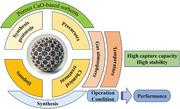

## 1 Introduction

Anthropogenic activities, especially the combustion of fossil fuels, emit substantial amounts of greenhouse gases into the atmosphere, contributing to global warming^[^ ^1^, ^2^, ^3^ ^]^ and an increase in extreme weather events.^[^ ^4^ ^]^ Therefore, deployment of carbon capture measures for the disposal of greenhouse gases is crucial for limiting emissions.^[^ ^5^ ^]^ Simultaneously, technological progress now facilitates the conversion of CO_2_ into value‐added chemicals, including starch,^[^ ^6^ ^]^ ethanol,^[^ ^7^ ^]^ gasoline,^[^ ^8^ ^]^ glucose,^[^ ^9^ ^]^ and formate,^[^ ^10^ ^]^ allowing CO_2_ to become vital carbon feedstock for the future with growing economic value. The development of cost‐effective carbon capture and storage (CCS) technology, aided by proposed policies of carbon pricing or carbon budgeting^[^ ^11^, ^12^ ^]^ presents great promise to help reach global ambitions of net‐zero CO_2_ emissions.^[^ ^13^ ^]^

The main CO_2_ capture technologies can be categorized into pre‐combustion (substituting carbonaceous fuel for carbon‐free alternatives such as H_2_),^[^ ^14^ ^]^ oxy‐fuel combustion (combusting carbonaceous fuel in purified oxygen to generate a pure stream of CO_2_)^[^ ^15^ ^]^ and post‐combustion techniques (separation of CO_2_ from diluent gases prior to exhausting to the atmosphere).^[^ ^16^ ^]^ Different CO_2_ sources, such as power plants,^[^ ^17^ ^]^ petroleum refineries,^[^ ^18^ ^]^ cement factory,^[^ ^19^ ^]^ steel mill,^[^ ^20^ ^]^ and chemical production^[^ ^20^ ^]^ are more or less suited to these three categories, and each require specific CO_2_ capture technology tailored to their process conditions and exhaust composition.

The most prominent post‐combustion CO_2_ separation methods are solution adsorption,^[^ ^21^ ^]^ membrane separation,^[^ ^22^ ^]^ solid absorption,^[^ ^23^, ^24^ ^]^ and chemical electrolyte separation.^[^ ^25^ ^]^ The use of solid sorbents helps overcome the high cost associated with the high enthalpy of vaporization and high reactivation energy of liquid adsorbents,^[^ ^26^ ^]^ while allowing for relatively high CO_2_ capture performance at high temperature (>500 °C), allowing for thermal integration with, e.g., power plants and steel production.^[^ ^27^, ^28^ ^]^ Solid oxide sorbents can be reversibly carbonated in CO_2_, and calcined in an inert atmosphere, via the equilibrium reaction *M*O + CO_2_ ↔ *M*CO_3_ (where ΔH_carbonation_ < 0), where *M* is an alkali metal or alkaline earth, with Ca and Mg being the most commonly applied elements.^[^ ^29^ ^]^ Naturally occurring^[^ ^30^, ^31^ ^]^ or synthetic^[^ ^32^ ^]^ materials can be used as sorbents, with a tradeoff between cost and performance: this review will focus on strategies for producing high performance synthetic sorbents, while minimizing additional cost relative to naturally occurring materials.

Calcium oxide‐based sorbents (with nominal capacity 0.78 g_CO2 _g^−1^_CaO_)^[^ ^33^ ^]^ can operate at 600–900 °C,^[^ ^34^ ^]^ and have attracted significant commercial interest as a post‐combustion technique in the power, chemical, and steel sectors.^[^ ^35^, ^36^, ^37^ ^]^ The high exothermicity of the carbonation reaction CaO + CO_2_ → CaCO_3_ (ΔH  =  ‐178 kJ mol^−1^, giving a volumetric energy density of 3.2 GJ m^−3^_CaO_) can also be applied as thermochemical energy storage in power plants to help manage heat and improve operating flexibility.^[^ ^38^, ^39^, ^40^ ^]^ The raw materials for producing CaO‐based sorbents are abundant in nature and non‐toxic, and relatively inexpensive (≈20$ tones^−1).[^ ^41^, ^42^ ^]^ Additionally, the waste from spent CaO‐based sorbents can be utilized in the cement industry.^[^ ^43^ ^]^

However, a major challenge in calcium looping (CaL) technology is the sintering of particles of CaO to form agglomerates, which can lead to a significant (up to 50%) decrease in CO_2_ capture capacity over multiple cycles, and hence extensive experimental efforts have been devoted to developing more stable sorbents by altering synthesis procedures and operational conditions.^[^ ^44^ ^]^ Despite these efforts, it remains challenging to ascertain whether the “optimal” results found in experiment represent the best achievable outcomes, given the vast number of degrees of freedom in operational parameters (e.g., temperature, CO₂ partial pressure, and cycle duration) and material properties (e.g., specific surface area and porosity).^[^ ^45^ ^]^

Therefore, machine learning (ML) strategies could be a promising alternative for designing and developing CaO‐based sorbents and optimizing the CaL process,^[^ ^46^ ^]^ such as linear regression, decision trees, and logistic regression, to predict material performance and properties with minimal mathematical formulation. Advanced models such as XGBoost can incorporate nonlinear features and complex interactions, capturing intricate relationships between material properties and performance metrics. By leveraging prior experimental data, ML models have the potential to accelerate the development of effective sorbents or catalysts;^[^ ^47^ ^]^ however, the reliability of these data‐driven approaches depends significantly on the quality and relevance of the input data.^[^ ^46^ ^]^ Statistically significant correlations between materials (e.g., synthesis methods and operational conditions) and performance metrics (e.g., CO₂ capture capacity and long‐term stability) are essential for ML models to yield useful insights for material development.

This paper reviews research from the past 10 years on CaO‐based sorbents, in particular, the influence of synthesis methods used (e.g., protocols and materials) and operational conditions on service stability and cyclic performance. By compiling a dataset of the synthesis methods, operational conditions, and cyclic performance of 1042 reported materials from literature (included in the Supporting Information), broad aggregate trends in the influence of different parameters were identified, described in Sections 2, 3, and 4. Section 5 then discusses kinetic models applied in literature for estimating rates of carbonation and calcination. Lastly, Section 6 considers the feasibility of several machine learning strategies to help to develop CaO‐based sorbents and operation processes, in order to achieve high effective carbon capture capacity and high stability.

## 2 Indices for Assessing the CaL Process

**Figure 1:**
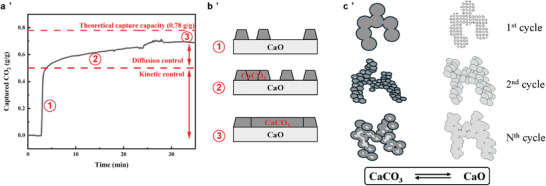
a) The change in reaction rate control regime during carbonation, b) the formation of a layer of CaCO_3_ on the CaO‐based sorbents during the carbonation process, c) schematic of the sintering effect over successive carbonation and calcination cycles.

In the carbonation process, CO_2_ reacts with CaO to form an external layer of CaCO_3_. When the thickness of the CaCO_3_ increases past a critical point (≈20–50 nm),^[^ ^48^ ^]^ the CaCO_3_ layer obstructs CO_2_ from diffusing to the surface to react with CaO, causing the effective reaction rate to shift from relatively rapid kinetic control to slow diffusion control (shown in **Figure** **1**a,b), reducing the overall effective rate of carbon capture, and making it difficult to achieve the theoretical CO_2_ capture capacity.

Another challenge is the sintering effect caused by repeated carbonation and calcination cycles, as shown in Figure 1c, as particles of CaO‐based sorbents tend to aggregate over time. Initially, the sorbents are present as small, uniform particles, but during the calcination process they break into smaller particles. During the subsequent carbonation process, the CaO‐based sorbents swell as a result of reaction with CO_2_, altering the overall structure. After multiple cycles, the sorbents tend to aggregate, leading to a decrease in specific surface area and porosity, which further decreases the available capture capacity. Hence, initial specific surface area, and decay in surface area per cycle, are key performance indices for assessing the net amount of CaO available for reaction with CO_2_ over time.

To enhance CaO‐based sorbents and optimize the CaL process, many researchers have studied altering both synthesis methods and operation conditions to mitigate the sintering effect. with key metrics such as CO₂ capture capacity applied to determine the performance of different sample.^[^ ^33^ ^]^ Thermogravimetric analysis (TGA) has been widely employed in mg‐scale experiments to estimate the CO₂ capture capacity (X_sorbent_) of sorbents, as described in **Equation** **1**, where m_carbN_ is the mass of CaO sorbents after carbonation, m_CalN_ is the mass of CaO‐based sorbents after calcination, and m_0_ is the mass of initial CaO‐based sorbents. For larger‐scale experiments (g‐scale and kg‐scale), this capture capacity can be determined using a gas analyzer at the outlet of a fixed‐bed reactor or fluidized‐bed reactor, as described in **Equation** **2**, where m_CO2_^in^ is the input mass flowrate of CO_2_, m_CO2_^out^ is the outlet mass flowrate of CO_2_, and t is the duration of one carbonation or calcination cycle.

(1) $$X_{sorbent}=\frac{m_{CarbN}-m_{CalN}}{m_{0}}$$

(2) $$X_{sorbent}=\frac{\int_{0}^{t}\left(m_{CO_{2}}^{in}-m_{CO_{2}}^{out}\right)dt}{m_{0}}\;$$

However, the capture capacity is affected by uncertainty in the initial mass of CaO relative to the total mass of the sorbent, which can include residual CaCO_3_ from incomplete calcination, and moisture as a result of incomplete conversion of CaO sorbents during the synthesis, as well as the loading of inert dopant materials.

Another indicator, decay rate,^[^ ^49^ ^]^ shown in **Equation** **3**, is used to determine the deactivation of the sorbents over repeated cycles, and hence the decrease in CO_2_ capture capacity. Lower values of the decay rate (ideally, 0) indicate high stability of the sorbents.

(3) $$Decay\;rate=\frac{X_{n}-\;X_{1}}{n}\;$$

where n is the cycle number, and X_1_, X_n_ are CO_2_ capture capacity at cycle 1 and cycle n, respectively.

Other important material parameters, such as mechanical strength, attrition resistance, and fluidization behavior were deemed beyond the scope of this study, but have been shown to influence the overall efficiency and operability of practical scale CaL processes.^[^ ^50^ ^]^

## 3 Performance Enhancement of CaO‐Based Sorbents

In this section, methods of enhancing CaO‐based sorbents to increase capacity and reduce the decay rate are discussed, including the protocols and materials used in synthesis to adjust the morphology and composition of particles of sorbent.^[^ ^51^, ^52^, ^53^, ^54^ ^]^

### 3.1 Synthesis Protocols

Common synthesis procedures include dry mixing (DM),^[^ ^55^, ^56^, ^57^ ^]^ wet mixing (WM),^[^ ^58^, ^59^, ^60^ ^]^ precipitation (PP),^[^ ^58^, ^59^, ^60^ ^]^ sol‐gel (SG),^[^ ^61^, ^62^, ^63^ ^]^ template (TP),^[^ ^64^, ^65^, ^66^, ^67^ ^]^ combustion (CB),^[^ ^68^, ^69^, ^70^ ^]^ impregnation (IM),^[^ ^71^, ^72^, ^73^ ^]^ and acid treatment (AT) methods.^[^ ^54^, ^74^, ^75^ ^]^ In all synthesis methods, precursors containing calcium are mixed with precursors containing other metals (as discussed in Section 3.4), to form a homogeneous CaO‐based product.

In the dry and wet mixing processes, metal oxide and carbonate precursors (i.e., CaO or CaCO_3_, and oxides or carbonates of any metal dopants) are crushed and mixed mechanically by ball milling^[^ ^76^ ^]^ or grinding^[^ ^77^ ^]^ (with the addition of a liquid binder to promote particle formation in the wet mixing process), followed by drying at ≈200 °C, and calcination at 700–1000 °C. In the precipitation technique, solutions of calcium salts and any other dopant metals are prepared and mixed. Then, the pH of the mixed solution is increased by adding, e.g., (NH_4_)_2_CO_3_ to the precipitate out particles of the sorbent. The precipitated particles are separated from the solution by centrifugation or filtration and then dried and calcined to remove residual ammonium ions.

Sol‐gel, template, and combustion methods are a group of similar techniques to one another, as they start by preparing a solution of calcium salts^[^ ^61^, ^62^, ^63^ ^]^ and an acid. The sol‐gel method condenses the solution under certain conditions to form a uniformly dispersed sol. The sol particles gradually connect to each other to form a 3D continuous network structure. After aging, the generated gel is dried and calcined, to generate a uniform porous structure. The template method uses a template material (e.g., starch,^[^ ^78^ ^]^ sucrose carbon,^[^ ^79^ ^]^ metal‐organic frameworks,^[^ ^80^ ^]^ or adsorbent paper)^[^ ^81^ ^]^ to absorb the salt solution., The template is then removed by calcination or chemical treatment, leaving pores with a specific microstructure, for example, hollow spheres.^[^ ^79^ ^]^ In the combustion synthesis method, the salt solution is mixed with a combustible material (e.g., urea^[^ ^82^ ^]^ or cigarette butts),^[^ ^83^, ^84^ ^]^ and is then dried and calcined. During the calcination process, the combustible materials ignite, rapidly releasing heat and gas, and hence facilitating generation of a porous structure in the final product.

In the impregnation procedure,^[^ ^71^, ^72^, ^73^ ^]^ particles of CaO or CaCO_3_ precursors are added to a solution of metal dopant salts (at an appropriate concentration to achieve the desired dopant loading). The resulting slurry is then dried to remove the water, and calcined to form particles of sorbent with a dopant or catalyst present at the surface of the particles.

In acid treatment methods, CaCO_3_‐based materials (either naturally derived, or synthesized using one of the methods described above) are washed with an acid, in order to roughen the surface of the material and increase pore volume.^[^ ^74^, ^85^, ^86^ ^]^ Acid treatment can also be applied to regenerate spent sorbents, in order to counteract loss of surface area as a result of sintering.^[^ ^87^ ^]^

As well as acid treatment, an alternative approach is to add a surfactant (e.g., ethanol) during synthesis,^[^ ^58^ ^]^ in order to enhance the affinity and permeability of H_2_O molecules to CaO, forming large pores as the water and ethanol are removed during drying and calcination.^[^ ^88^ ^]^

In addition to the relatively well‐established approaches described above, other novel sorbent preparation methods (OT) including electrospinning^[^ ^89^ ^]^ and atomic layer deposition^[^ ^90^ ^]^ have been investigated at laboratory scale, but their complexity limits their use in industrial applications.

**Figure 2:**
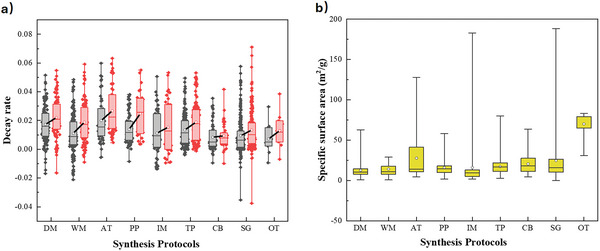
Distributions of a) decay rate and b) specific surface area for CaO‐based sorbents, based on a survey of ≈200 recent papers. The lower and upper edges of each box correspond to the first and third quartile values of each data set respectively, with the horizontal line indicating the median value; whiskers indicate the maximum and minimum reported values. The black and red boxes in (a) show the average deactivation of sorbents after 10 and 20 cycles, respectively, with a solid black line connecting the mean values (white circles).

From a literature survey of ≈200 papers related to the synthesis of CaO‐based sorbents from the past ≈10 years using the techniques described above, corresponding to 1042 reported materials and conditions, the distributions of reported decay rate and specific surface are plotted in **Figure** **2** (with the full dataset tabulated in the Supporting Information). Negative values of decay rate are a mathematical artefact corresponding to samples which showed an initial improvement in capacity over the first 2–3 cycles^[^ ^69^ ^]^ followed by decay in subsequent cycles, resulting in a net increase in capacity between the first, and 10th, or 20th cycles. An increase in sorbent capacity over the first few CaL cycles (also referred to as ‘self‐reactivation) occurs as a result of a porous structure developing as CO_2_ is removed during calcination, exposing new internal areas of the material during the subsequent carbonation step.^[^ ^53^, ^91^ ^]^

In Figure 2a, the change in median decay rate after 10 and 20 cycles is indicated by a diagonal black line, with a steep gradient indicating a relatively rapid increase in rate of decay. Hence, samples prepared using combustion, impregnation, template, and sol‐gel methods showed the shallower gradient for increase in decay rate, and therefore the highest cyclic stability. Furthermore, Figure 2b shows that samples with the highest specific surface area were achieved using the acid treatment, impregnation, and sol‐gel methods, albeit with relatively little difference in average values between methods, with the exception of novel, costly methods such as electrospinning or atomic layer deposition (OT). Such methods were able to achieve considerably higher values of specific surface area, but are currently prohibitively expensive for practical industrial applications.

**Figure 3:**
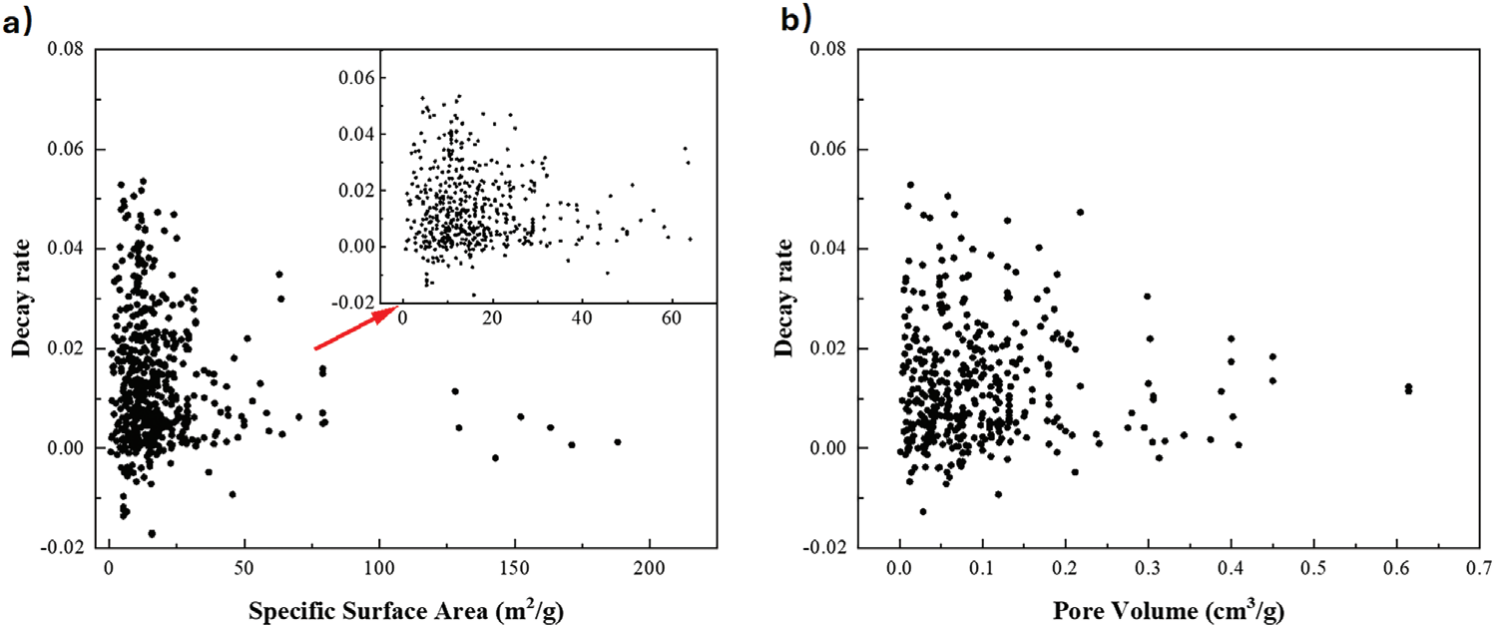
The relationships between decay rate over 10 cycles and a) specific surface area and b) specific pore volume for CaO‐based sorbents reported in literature.

Reported values of decay rate, specific surface area, and specific pore volume from literature are plotted in **Figure** **3**. For both surface area and pore volume, the reported values form an approximately triangular‐shaped distribution:, i.e., materials with low surface area or pore volume showed a broad range of decay rates, whereas samples with the highest surface area or pore volume showed lower decay rates, consistent with experimental studies indicating a correlation between microporosity and decreased activity loss.^[^ ^92^ ^]^

**Figure 4:**
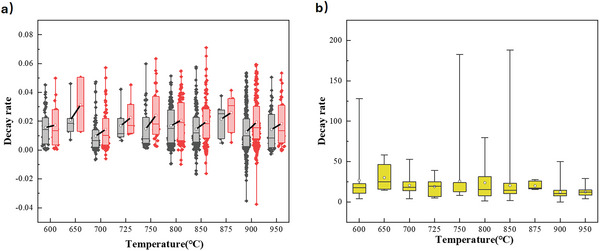
The effect of calcination temperature on a) decay rate, and b) specific surface area. The lower and upper edges of each box correspond to the first and third quartile values of each data set respectively, with the horizontal line indicating the median value; whiskers indicate the maximum and minimum reported values. The black and red boxes in (a) show the average deactivation of sorbents after 10 and 20 cycles, respectively, with a solid black line connecting the mean values (white circles).

Another parameter with a considerable impact on material properties is the temperature applied for calcination of the precursors during synthesis (as distinct from the calcination conditions applied during CaL operation),^[^ ^56^, ^69^, ^93^, ^94^, ^95^ ^]^ with the distributions in reported decay rate and surface area shown in **Figure** **4**.

Previous work has reported that calcination temperatures above ≈750 °C facilitate formation of a porous structure, whereas higher temperatures (>900 °C) can cause particle sintering during synthesis, decreasing overall surface area.^[^ ^69^, ^93^, ^94^ ^]^ However, it is difficult to systematically assess the effect of calcination temperature on overall properties (as indicated by the limited differences in distribution observed between conditions in Figure 4), as a result of the large number of other degrees of freedom applied during calcination (duration, heating and cooling rates, gas environment, number of calcination stages, etc.). Additionally, for synthesis methods applying an organic template or combustible material, increased calcination time relative to other methods is required to ensure complete burnout of the template molecule,^[^ ^95^ ^]^ although further calcination after complete burnout would be unlikely to improve material properties further.

Additionally, other parameters during synthesis affect stability and material properties. Dervin and Pillai^[^ ^96^ ^]^ reported a significant impact of ageing time during sol‐gel synthesis, with longer ageing times resulting in greater mechanical strength of products and higher pore volume. Song^[^ ^97^ ^]^ investigated the effect of different drying methods (e.g., spray drying, evaporation drying, and freeze drying) on the structure of CaO‐based sorbents. Spray drying produced sorbents with superior performance, as materials produced using the other methods showed poor mechanical stability, as a result of material contraction caused by evaporation, and the soft ‘fluffy’ structure formed during freezing

### 3.2 Precursors

**Figure 5:**
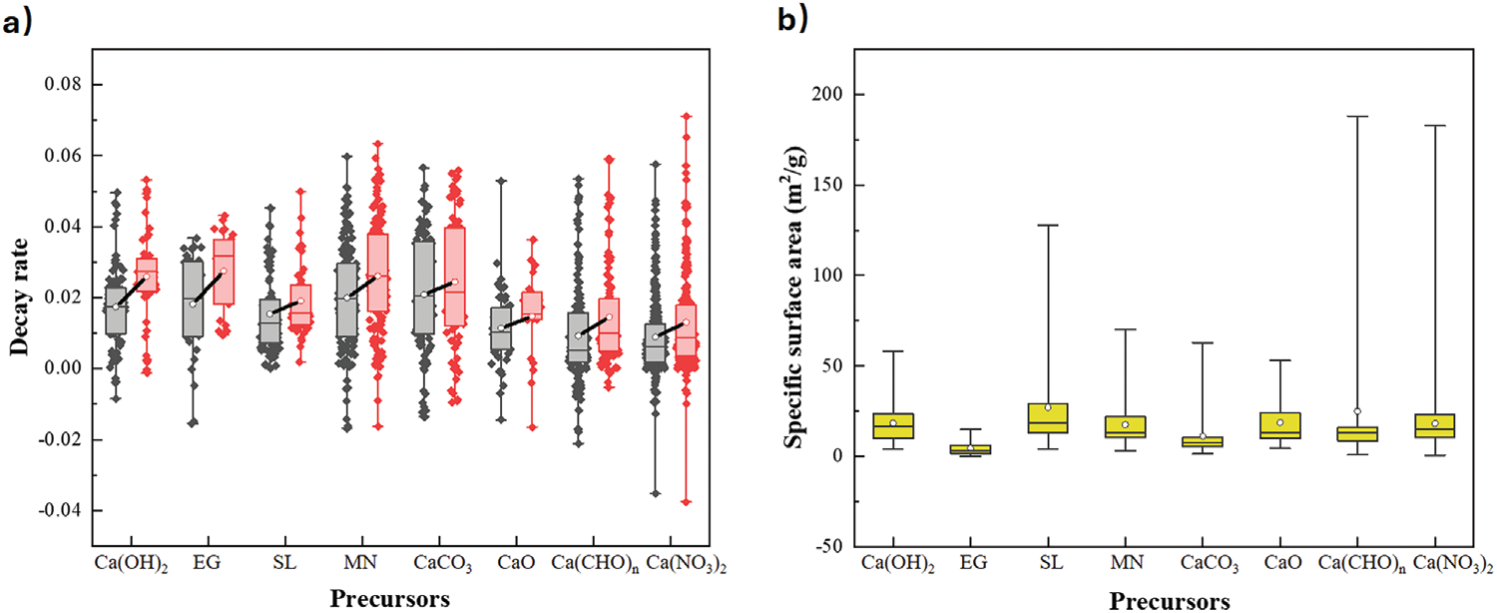
a) The effect of calcium precursors used on a) decay rate, and b) specific surface area. The lower and upper edges of each box correspond to the first and third quartile values of each data set respectively, with the horizontal line indicating the median value; whiskers indicate the maximum and minimum reported values. The black and red boxes in (a) show the average deactivation of sorbents after 10 and 20 cycles, respectively, with a solid black line connecting the mean values (white circles).

The choice of calcium precursors also affects the performance of sorbents, with common precursors including naturally occurring minerals (MN) (e.g., limestone^[^ ^98^, ^99^ ^]^ and dolomite),^[^ ^100^ ^]^ eggshells (EG),^[^ ^72^ ^]^ industrial slag (SL),^[^ ^101^, ^102^, ^103^ ^]^ partially refined minerals such as calcium carbonate (CaCO_3_)^[^ ^73^ ^]^ or calcium hydroxide (Ca(OH)_2_),^[^ ^104^ ^]^ and synthetic precursors such as calcium nitrate Ca(NO_3_)_2_^[^ ^66^ ^]^ or organic calcium salts (Ca(CHO)_n_).^[^ ^105^, ^106^ ^]^ **Figure** **5** illustrates the effect of precursors on the stability and specific surface area of the resulting CaO‐based sorbents. Sorbents prepared using synthetic calcium salts, especially calcium nitrate, show the highest stability and specific surface area, because decomposition of the anions during the calcination stage can help generate microporous structures.^[^ ^107^, ^108^ ^]^

### 3.3 Chemical Treatment

For sorbents prepared using acid treatment methods, the influence of the choice of acid used on material properties has been widely investigated.^[^ ^85^ ^]^

**Figure 6:**
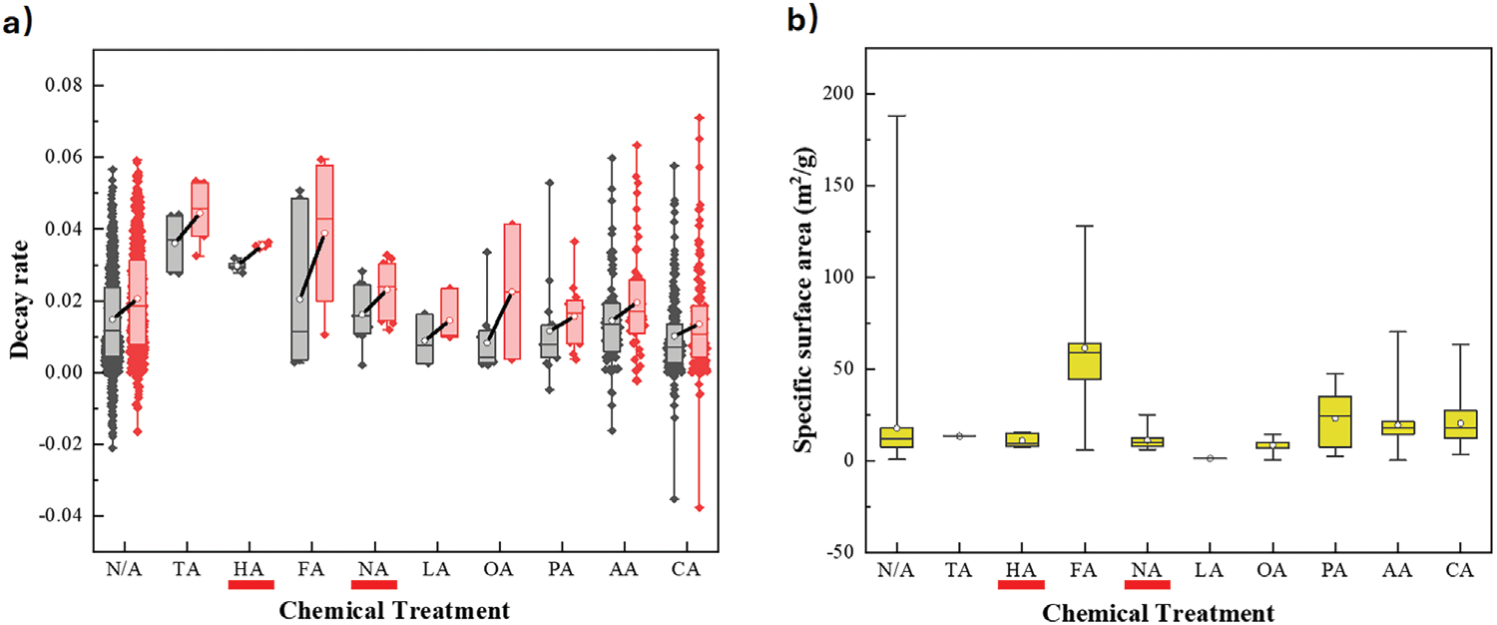
The effect of different acids during acid treatment on a) decay rate, and b) specific surface area. The lower and upper edges of each box correspond to the first and third quartile values of each data set respectively, with the horizontal line indicating the median value; whiskers indicate the maximum and minimum reported values. The black and red boxes in (a) show the average deactivation of sorbents after 10 and 20 cycles, respectively, with a solid black line connecting the mean values (white circles). Mineral acids (hydrochloric acid (HCl, HA) and nitric acid (HNO_3_, NA)) are underlined in red.

The effects of treatment with organic acids including formic acid (FA),^[^ ^109^ ^]^ acetic acid (AA),^[^ ^110^ ^]^ propionic acid (PA),^[^ ^68^ ^]^ citric acid (CA),^[^ ^63^ ^]^ oxalic acid (OA),^[^ ^110^ ^]^ lactic acid (LA,^[^ ^110^ ^]^ and tartaric acid (TA,^[^ ^75^ ^]^ and mineral acids (e.g., nitric acid (NA),^[^ ^109^ ^]^ and hydrochloric acid (HA)),^[^ ^109^ ^]^ are summarized in **Figure** **6**. In general, organic acids showed superior performance to mineral acids in increasing surface area, and hence cyclic stability. During treatment with an organic acid, the acid reacts with the calcium precursor to form a xerogel. Then, during calcination, the organic calcium salt decomposes, resulting in a rapid drop in molecular weight and an increase in local density, resulting in a porous structure, in a manner similar to organic template methods.^[^ ^75^, ^86^ ^]^ However, Soares Dias et al.^[^ ^97^ ^]^ found that while treatment with relatively low molecular weight acids, especially citric acid, helped promote high surface area by forming a xerogel, the heat released during burnout of larger molecules (e.g., tartaric acid) promoted sintering, and thus negatively impacted the stability of the sorbents. Moreover, citric acid acts as a chelating agent during acid treatment, promoting the generation of small, uniform nanoparticles of CaO^[^ ^111^ ^]^ after calcination.

However, despite samples treated with some organic acids showing a greater average surface area (in particular, FA, PA, AA, and CA), the differences in cyclic stability between treatment methods were limited. Therefore, differences in chemical treatment were deemed unlikely to be the determining factor when selecting conditions for sorbent preparation.

### 3.4 Dopants

The cyclic stability of CaO‐based sorbents can be improved by adding dopants with high Tamman temperatures (i.e., the minimum temperature required for the atoms in the bulk material to become mobile). Under typical conditions, the CaL process operates well above the Tamman temperature of CaCO_3_ (533 °C), rendering some degree of sintering inevitable for pure CaO‐CaCO_3_.^[^ ^112^ ^]^ Dopants with a Tamman temperature higher than the calcination temperature (>700‐900 °C) are able to act as an inert refractory “spacer” to prevent aggregation of CaCO_3_‐CaO particles,^[^ ^49^ ^]^ with examples of possible dopants given in **Table** **1**.

**Table 1:** The Tammann temperatures and melting temperatures of some metal oxides investigated as structural dopants to mitigate sintering.

Metal Oxide	Tammann temperature [°C]	Melting temperature [°C]	Refs.
MgO	1276	2852	[123]
ZrO_2_	1218	2715	[124]
CeO_2_	1064	2600	[124]
Y_2_O_3_	1200	2425	[118]
Yb_2_O_3_	–	2435	[125]
La_2_O_3_	1013	2315	[118]
Nd_2_O_3_	–	2233	[126]
Al_2_O_3_	891	2051	[123]
Pr_6_O_11_	–	2042	[126]
ZnO	710	1975	[118]
MnO	962	1945	[127]
CoO	779	1935	[124]
BaO	982	1923	[118]
Ga_2_O_3_	–	1900	[126]
TiO_2_	785	1834	[124]
SiO_2_	725	1710	[124]
Fe_2_O_3_	698	1565	[118]
CuO	526	1326	[128]
MoO_3_	534	795	[129]

A selection of other elements has also been investigated for modified CaO‐based sorbents, primarily as electronic dopants to improve surface affinity to CO_2_ and hence improve absorption kinetics. Examples of such dopants include rare earth elements (La,^[^ ^72^ ^]^ Ce,^[^ ^113^ ^]^ Pr,^[^ ^49^ ^]^ Nd,^[^ ^114^ ^]^ Yb),^[^ ^115^ ^]^), alkali metals and alkaline earths (Li,^[^ ^116^ ^]^ Na,^[^ ^73^ ^]^ K,^[^ ^71^ ^]^ Mg,^[^ ^53^ ^]^ Ca,^[^ ^117^ ^]^ Ba),^[^ ^118^ ^]^ transition metals (Y,^[^ ^53^ ^]^ Mn,^[^ ^119^ ^]^ Fe,^[^ ^81^ ^]^ Co,^[^ ^118^ ^]^ Cu,^[^ ^105^ ^]^ Zn,^[^ ^118^ ^]^ Zr,^[^ ^65^ ^]^ Mo),^[^ ^65^ ^]^ and post‐transition metals and metalloids (Al,^[^ ^94^ ^]^ Ga,^[^ ^120^ ^]^ Si).^[^ ^121^ ^]^

Some industrial waste materials, such as spent bleaching clay, which contains a mixture of metal ions and combustible components, have also been applied as dopants, increasing specific surface area and pore volume^[^ ^122^ ^]^ via combustion synthesis, as well as depositing metal ions at the surface of the sorbent.

**Figure 7:**
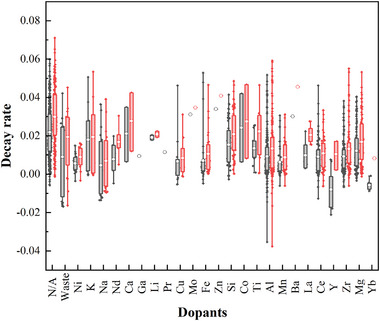
The effect of dopants on the stability of CaO‐based sorbents. The lower and upper edges of each box correspond to the first and third quartile values of each data set respectively, with the horizontal line indicating the median value and white circle indicating the mean values; whiskers indicate the maximum and minimum reported values. The black and red boxes show the average deactivation of sorbents after 10 and 20 cycles, respectively.

As shown in **Figure** **7**, CaO‐based sorbents doped with metal oxides with relatively high Tammann temperature (>900 °C, e.g. MgO, ZrO_2_, CeO_2_, Y_2_O_3_) are clustered at relatively low decay rates, and hence high cyclic stability. Contrastingly, some materials doped with metal oxides with relatively low Tamman temperatures (<750 °C, e.g. Zn, Mo, Co) show poor performance, as the dopants accelerate grain boundary movement and block pore structure.^[^ ^49^ ^]^ However, some oxides with relatively low Tamman temperatures (e.g., Al_2_O_3_, SiO_2_, CuO) show better performance than might be initially expected, as a result of forming stabilized coordination complexes with CaO (e.g., Ca_9_Al_6_O_18_^[^ ^130^ ^]^ and CaSiO_4_),^[^ ^131^ ^]^ which inhibit grain growth of CaO^[^ ^104^ ^]^ and hence mitigate sintering.

Additionally, alkali metal and alkaline earth metal salts with relative low melting temperature (600‐950 °C), including NaCl,^[^ ^132^ ^]^ KCl,^[^ ^133^ ^]^ CaCl_2_,^[^ ^134^ ^]^ Na_2_CO_3_,^[^ ^135^, ^136^ ^]^ Na_2_SO_4_,^[^ ^132^, ^137^ ^]^ K_2_CO_3_,^[^ ^138^ ^]^ KMnO_4_,^[^ ^139^ ^]^ and CaBr_2_^[^ ^140^ ^]^ have been shown to enhance CaO‐based sorbents^[^ ^71^ ^]^ by acting as surface catalysts for calcination, allowing for operation at reduced temperature.^[^ ^141^ ^]^ However, Gonzalez et al.^[^ ^142^ ^]^ report that while alkali metals might be beneficial as surface catalysts, some alkali metal salts can promote sintering of CaO grains.

Furthermore, doping with metal oxides with variable valence state, such as Fe and Cu, can act as catalysts by providing additional oxygen vacancies to facilitate the mechanism of CO_2_ capture.^[^ ^62^ ^]^

**Figure 8:**
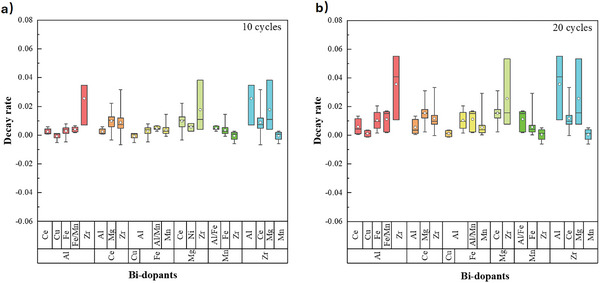
The effect of bi‐dopants on the stability of CaO‐based sorbents after a) 10 cycles, and b) 20 cycles.

As well as doping with single elements, various studies have investigated applying combinations of metal dopants (shown in **Figure** **8**), where second metal alters the microscopic morphology and structural characteristics of the CaO‐based sorbents, generating a composite oxide with a higher temperature resistance.^[^ ^81^ ^]^ The combinations Fe/Mn, Mn/Zr, Al/Cu, and Al/Ce/Zr all showed promising performance after 10–20 cycles. However, the addition of a large mass fraction of dopants necessarily decreases the specific carbon dioxide capacity of a given sorbent, as the mass fraction of CaO is decreased, and therefore a greater total mass of solids is required for equivalent capacity.

### 3.5 Storage and Transport

In addition to the synthesis parameters discussed above, storage conditions can impact the performance of CaO‐based sorbents. During prolonged storage in air, CaO can react with ambient moisture to form Ca(OH)_2_.^[^ ^143^ ^]^ Kim et al.^[^ ^143^ ^]^ and Liang et al.^[^ ^104^ ^]^ found that for CaO‐based sorbents stabilized with Ca_9_Al_6_O_18_ as a dopant, the presence of Ca(OH)_2_ promoted the decomposition of Ca_9_Al_6_O_18_ to Ca_12_Al_14_O_33_, decreasing cyclic stability relative to samples that had not been exposed to moisture. Therefore, in order to preserve the stabilizing effect of any calcium aluminate dopants present, CaO‐based sorbents should be stored and transported in the less hydroscopic carbonated state, and then calcined for use in the CaL process.^[^ ^104^ ^]^

## 4 The Effects of Carbonation and Calcination Process Conditions for CaL

Another approach for optimizing CaL systems is to adjust the operating conditions, in particular the temperatures and gas atmospheres in the carbonation and calcination stages.^[^ ^144^, ^145^ ^]^

**Figure 9:**
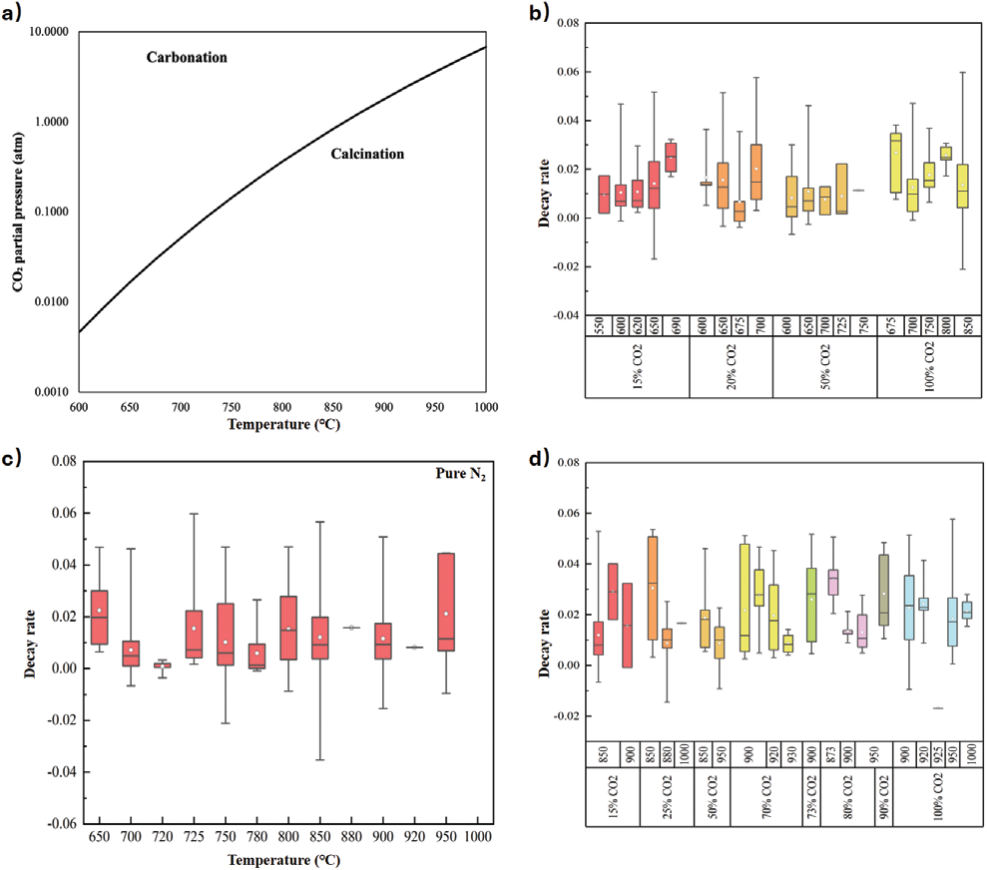
a) Representative phase diagram for the equilibrium reaction CaO + CO_2_ ↔ CaCO_3_, generated using FactSage software,^[^ ^139^ ^]^ b) The effect of carbonation temperature on the stability of CaO‐based sorbents, c) The effect of calcination temperature in inert gas on cyclic stability and d) The effect of the calcination temperature in varying CO_2_ environments on cyclic stability. The lower and upper edges of each box in (b–d) correspond to the first and third quartile values of each data set respectively, with the horizontal line indicating the median value and white circle indicating the mean; whiskers indicate the maximum and minimum reported values.

**Figure** **9a** illustrates the change in CO_2_ equilibrium partial pressure (*pCO_2,eq_*) for the carbonation and calcination of CaO‐CaCO_3_ at different temperatures. In order to successfully achieve cyclic carbonation and decarbonation, the conditions (in terms of temperature and CO_2_ partial pressure) in the carbonation and calcination reactors must be above and below the equilibrium line, respectively. Furthermore, for a real industrial CaL process, the partial pressure of CO_2_ in the feed to the carbonation stage is likely to be determined by the upstream process conditions, thereby setting the maximum equilibrium temperature, *T_eq_*, for carbonation. For example, flue gas from a typical coal fired power plant might contain *pCO_2_ = ≈*0.1 atm (*T_eq_ =* ≈730 °C),^[^ ^146^, ^147^ ^]^ whereas flue gas from a cement plant can reach up to *pCO_2_ =* ≈0.3 atm (*T_eq_ =* ≈790 °C),^[^ ^148^, ^149^ ^]^ or, in an extreme case, direct CO_2_ capture from air has *pCO_2_* = ≈420 × 10^−6^ atm^[^ ^150^ ^]^ (*T_eq_ =* ≈520 °C).

Therefore, all else being equal, operating at the lowest thermodynamically feasible temperature for both steps would appear to be desirable for maintaining high cyclic stability, in order to mitigate sintering during calcination,^[^ ^151^, ^152^ ^]^ and to maximise the thermodynamic driving force for carbonation. However, at lower temperatures (<700 °C), kinetic limitation can cause a decline in the amount of CO_2_ captured per cycle due to incomplete calcination, resulting in a build‐up of unreacted CaCO_3_ in the sorbents over repeated cycles. As such, intermediate reaction temperatures can provide improved cyclic stability by balancing reaction kinetics against physical degradation, with a further description of the kinetics of carbonation and calcination given in Section 5. For example, Ma et al.^[^ ^56^ ^]^ found that for a CaO‐based sorbent doped with Fe, carbonation at 750 °C and *pCO_2_* = 0.25 atm showed greater CO_2_ uptake per cycle over 20 cycles than samples carbonated at 650 °C or 800 °C, with calcination temperature fixed at 750 °C. Despite exceeding the theoretical equilibrium temperature for carbonation at *pCO_2_* = 0.25 atm (*T_eq_* = 780 °C), the authors do report some CO_2_ uptake at 800 °C, possibly as a result of the Fe dopant altering the thermodynamic characteristics of the material relative to pure CaO. Similarly, during calcination, the authors also found that a calcination temperature of 880 °C gave improved cyclic stability as compared to calcination at 1000 °C, where sintering resulted in rapid degradation to <60% of the initial sorbent capacity over 20 cycles. However, the material showed a similar rate of decay in cyclic capacity for calcination at 880 °C with *pCO_2_ =* 0 atm or 0.25 atm, indicating that, aside from setting the minimum temperature required for calcination (or, equivalently, the maximum feasible temperature for carbonation), the absolute concentration of CO_2_ does not appear to have a strong impact on the degradation of CaO‐based sorbents with repeated cycles. Moreover, higher *pCO_2_* conditions during calcination might be desirable in order to decrease subsequent compression requirements^[^ ^153^, ^154^ ^]^ for CO_2_ transport and sequestration (generally to ≈150 atm).^[^ ^155^, ^156^ ^]^

Figure 9b–d show the reported decay rates for different materials, at different combinations of *pCO_2_* and temperature for carbonation (Figure 9b) and calcination (Figure 9c,d). No clear trend was observed with respect to temperature at each *pCO_2_* condition, despite, for each given material, decay rate being expected to be most severe at very high (>900 °C)^[^ ^56^, ^144^ ^]^ or low (<700 °C) temperatures as a result of sintering and incomplete calcination, respectively. However, the differences in reaction kinetics and sintering resistance between materials as a result of the synthesis steps described in Section 3 mean that for the whole dataset, differences in behaviour with respect to temperature approximately cancelled out. Therefore, in order to achieve an optimal balance between thermodynamically feasible carbonation for a given feed stream, rapid kinetics of carbonation and calcination, and limited sintering, the target operating conditions should be tailored to each material and process, with an empirical operating range of ≈600–900 °C,^[^ ^157^, ^158^ ^]^ rather than a particular temperature emerging as optimal for all materials and systems.

Some research has also investigated the use of steam during calcination and carbonation to aid CO_2_ desorption and absorption respectively, by facilitating diffusion of CO_2_ into the sorbent^[^ ^159^ ^]^ and promoting pore formation during calcination, increasing total capacity by ≈10wt.%.^[^ ^160^, ^161^ ^]^ However, at >800 °C, the presence of steam was found to promote grain growth of CaO crystals,^[^ ^159^, ^162^ ^]^ promoting sintering and partially offsetting any improvement in uptake capacity.

## 5 Kinetic Models for Carbonation and Calcination

### 5.1 The Random Pore Model of Carbonation

In order to model the behavior of the carbonation reaction CaO + CO_2_ → CaCO_3_, a variety of kinetic models have been employed, including the grain model (GM),^[^ ^163^, ^164^ ^]^ random pore model (RPM),^[^ ^165^, ^166^, ^167^ ^]^ and shrinking core model (SCM),^[^ ^164^ ^]^ with the random pore model providing the most accurate alignment with experimental measurements.^[^ ^167^ ^]^

The RPM model considers the effect of initial specific surface area, porosity, and pore length on conversion as follows:^[^ ^165^ ^]^

(4) $$X\;\left(\tau \right)=1-{\left(1-\frac{\tau }{\sigma }\right)}^{3}\exp\left(-\tau \left(1+\frac{\psi \tau }{4}\right)\right)$$

where τ is dimensionless time, and σ and Ψ are dimensionless parameters corresponding to particle size and structure, given below

(5) $$\tau \;=\frac{rS_{0}t}{1-\varepsilon_{0}}\;$$

(6) $$\sigma =\frac{d_{p}S_{0}}{2\left(1-\varepsilon_{0}\right)}\;$$

(7) $$\psi =\frac{4\pi L_{0}\left(1-\varepsilon_{0}\right)}{{S_{0}}^{2}}\;$$

where *d_p_* is the particle diameter (m^2^), *L_0_* is the initial total pore length (m^2^), *S_0_* is the initial surface area available for reaction per unit volume (m^−1^), and ε *_0_* is the initial porosity. Values of *L_0_*, *S_0_*, and ε *_0_* are determined for a given sample using porosimetry measurements, e.g., N_2_ physisorption or Hg porosimetry.^[^ ^167^ ^]^

Based on this initial equation, Scaltsoyiannes et al.^[^ ^167^ ^]^ improved the random pore model to incorporate inert dopants as discussed in Section 3.4, adjusting the values of *L_0_*, *S_0_*, and ε *_0_* with respect to the volume fraction of pure CaO in the calcined material (i.e., such that ε *_0_^CaO^* corresponds to the volume fraction of inert material plus empty space). In order to adjust *S_0_* to account for the fraction of inert material, the size ratio of crystallites in the CaO and inert phases(s) must be known and can be determined from, e.g., X‐ray diffraction measurements.

However, as the initial structure of the sorbents will change over repeated carbonation and calcination cycles, the initial values of *L_0_*, *S_0_*, and ε *_0_* measured from fresh samples might not be accurate at the start of successive cycles, as the RPM does not account for material degradation. Therefore, although the RPM provides a reasonably accurate description of the kinetics of carbonation for the first cycle of a CaL process for a given material, the estimated conversion from the RPM is likely to be an overestimate for later cycles. Furthermore, for the modified RPM equation incorporating inert solid phases,^[^ ^167^ ^]^ the ratio of crystallite sizes between each phase is likely to change as the material sinters, resulting in further deviation. To achieve more accurate estimates of *L_0_*, *S_0_*, and ε *_0_* for later cycles, samples of the CaO‐based sorbent can be periodically withdrawn from the carbonation reactor, and the characterization measurements repeated, in order to track the approximate change per cycle in each parameter.

### 5.2 Models for CaCO_3_ Calcination

The calcination of CaCO_3_ to form CaO can be described by a shrinking‐core model,^[^ ^168^, ^169^, ^170^ ^]^ of the form given in **Equation** **8**, where *r* is the velocity of the reaction front into the particle, *k*_1_ is a rate constant (m4 mol^−1^s^−1^), *R* is the molar gas constant (kJ mol^−1^K^−1^), *T* is the reaction temperature (K), *P_CO2_* is the partial pressure of CO_2_ (Pa), and *P_e_* is the equilibrium partial pressure of CO_2_ (Pa), determined from tables of thermodynamic data (as plotted in Figure 9a) or estimated from an empirical relationship, such as the one given in Equation 9.^[^ ^168^ ^]^ The variation of the rate constant with temperature is given by the Arrhenius relation, as shown in Equation 10, where *A* is the pre‐exponential factor (m4 mol^−1^ s^−1^) and E_a_ is the activation energy (kJ mol^−1^)

(8) $$r\;\left(T,C_{\mathrm{CO}2}\right)=k_{1}\;\frac{1}{\mathrm{RT}}\left(P_{\mathrm{CO}2}-P_{e}\right)$$

(9) $$P_{e}\approx 4.083\times 10^{7}\times \exp\left(-\frac{20474}{T}\right)$$

(10) $$k_{1}=\;A\times \exp\left(-\frac{E_{a}}{\mathrm{RT}}\right)$$

As discussed in Section 3, the effective CO_2_ capacity of CaCO_3_ is affected by the pore structure of the material, which changes as the material sinters. Shi et al.^[^ ^169^ ^]^ attempted to directly model the changes in pore structure for samples of limestone during limestone, with reasonable agreement between calculated and measured specific surface area over the course of a calcination cycle. However, in order to approximate the effective CO_2_ capacity of a given sorbent over repeated cycles of calcination and carbonation, the correlation reported by Grasa and Abanades^[^ ^171^ ^]^ (**Equation** **11**) can be applied, where a_N_ is the maximum conversion of the sorbent in cycle *N*, *a_∞_* is the residual (final) conversion of the sorbent after a very large number of cycles, and *k_decay_* is a fitted parameter for the material of interest.

(11) $$a_{N}=\left(\frac{1}{\frac{1}{\left(1-a_{\infty }\right)}+k_{decay}N}\right)+a_{\infty }$$

However, the kinetic models for carbonation and calcination, and the empirical model for decay in capacity over repeated cycles, reported in this section require experimental measurements in order to fit parameters, with the values of fitting parameters being affected by the synthesis parameters discussed in Section 3 and the operation parameters discussed in Section 4. Therefore, in order to predict the performance of novel sorbents for CaL without the need for lengthy characterization experiments, machine learning approaches could offer a promising opportunity to develop a model linking synthesis and process parameters to effective CO_2_ capacity and cyclic stability, discussed in Section 6.

## 6 Possible Machine Learning Strategies for Improving CaL Materials and Processes

Machine learning (ML) methods are essentially a “black box” for applying a specific set of nonlinear or linear functions to link input measurements to output predictions. For typical research in materials science, there are often complex relationships between conditioning factors (e.g., synthesis parameters) and target properties that are difficult to handle with traditional, non‐ML based analytical methods. Machine learning models can be separated into three main categories: regression, classification & clustering, and probability estimation. Probabilistic estimation algorithms are mainly used for the discovery of new materials,^[^ ^44^, ^172^ ^]^ while regression, and classification & clustering algorithms are used for the prediction of material properties at the macro and micro levels,^[^ ^44^ ^]^ without considering the intrinsic physical principles governing reactive behavior.

**Figure 10:**
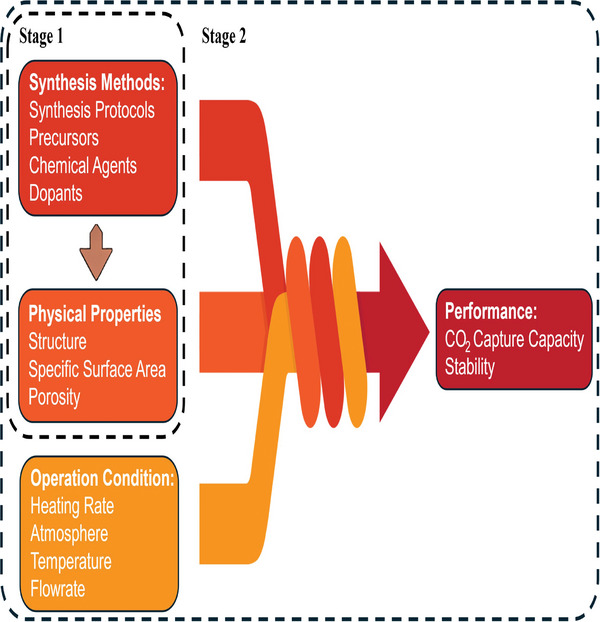
Schematic representing the input and output data for a two‐stage prediction model based on machine learning. In stage 1, the synthesis protocol is used to estimate material properties, and then in stage two, both are combined with the reactor operating conditions to estimate the overall sorbent performance.

The material property dataset collected from literature in this review (presented in the Supporting Information) is suitable for tubular dataset regression, as the data structures are arranged in the form of tables. Through regression analysis, the relationship between material properties and target performance can be learned from existing data, thereby predicting the performance of new materials, or optimizing the synthesis method of existing materials. The machine learning methods applicable for this tabular dataset can be separated into two broad groups: deep neural networks and tree‐based machine learning models.^[^ ^173^, ^174^ ^]^

As discussed in the preceding sections, four principal factors form the dataset collected in this review, namely synthesis materials and protocol, physical material properties, operation condition and reactive performance, with relevant sub‐categories for each given in **Table** **2**.

**Table 2:** Material features compiled in this review, with variable type (categorical or numerical) indicated.

Category	Sub‐category	Name	Variable Type
Synthesis	Material	Calcium core	Categorical
		Molar mass of calcium core	Numerical
		Dopants	Categorical
		Molar mass of dopants	Numerical
		Template	Categorical
		Molar mass of templates	Numerical
		Acid	Categorical
		Molar mass of acid	Numerical
	Method	Protocols	Categorical
	Calcination conditions	Temperature [°C]	Numerical
		Heating rate [°Cmin^−1^]	Numerical
		Gas	Categorical
		Time (min)	Numerical
Physical properties		Surface area [m^2 ^g^−1^]	Numerical
		Pore volume [cm^3 ^g^−1^]	Numerical
		Pore Size [nm]	Numerical
		Structure of sorbents	Categorical/ Graphic
Operation conditions	Carbonation stage	Temperature (°C)	Numerical
		Gas atmosphere	Categorical
		Heating rate (°C Min^−1^)	Numerical
		Time (min)	Numerical
	Calcination stage	Temperature (°C)	Numerical
		Gas atmosphere	Categorical
		Heating rate (°C Min^−1^	Numerical
		Time (min)	Numerical
Reactive performance		Capture capacity [g g^−1^]	Numerical
		Stability [%]	Numerical

Effective carbon capture capacity and cyclic stability are the key performance indicators for assessing CaO sorbents, as discussed in Section 2, and are influenced by material characteristics, including specific surface area and porosity. These characteristics, in turn, depend on synthesis variables, such as protocols, precursors, dopants, and chemical treatments.

The strong correlation between synthesis methods, physical properties, and reactive performance, as discussed in Sections 3 and 4, renders them suitable parameters for applying machine learning strategies. (**Figure** **10**)

### 6.1 Deep Neural Networks

With the rise of deep learning,^[^ ^175^ ^]^ numerous neural network architectures have been adapted to handle tabular data effectively. These deep learning methods can be further divided into differentiable trees, convolutional neural networks (CNNs), attention mechanisms, regularization methods, and explicit modeling of multiplicative interactions.

Differentiable trees^[^ ^176^, ^177^, ^178^ ^]^ aim to make conventional decision trees compatible with gradient‐based learning (i.e., where the rate of change of each function is estimated with respect to each parameter in order to iteratively improve the model) by introducing differentiable splits. Unlike conventional trees with hard splits between binary outcomes, differentiable trees use soft, learnable splits that allow the model to propagate gradients throughout the structure. The Neural Oblivious Decision Ensembles (NODE)^[^ ^178^ ^]^ method is an example in this category, where each decision node assigns weights to features instead of making binary choices, making the model compatible with backpropagation and improving flexibility in deep learning frameworks.

Convolutional Neural Networks (CNNs) are particularly well‐suited for image data. The core building block of CNNs, convolutional layers,^[^ ^179^, ^180^ ^]^ which have shown impressive results for feature extraction in image and audio data, have also been adapted for 1D‐CNN architectures that operate on tabular data.^[^ ^181^ ^]^ These models apply 1D convolutions across features, enabling local feature interactions within the input. However, unlike images, tabular data features often lack locality (i.e., relationships between variables based on proximity with the dataset) or spatial ordering, which CNNs typically rely on. To overcome this, some models use fully connected layers (i.e., where every neuron in the model is connected to every neuron in the next layer, such that global relationships can be identified) to expand feature representations, creating new feature sets with artificial locality to make the convolutions more effective.^[^ ^181^ ^]^ However, the use of fully connected layers leads to a large number of fitting parameters, making the model computationally expensive and prone to overfitting.

Attention mechanisms,^[^ ^182^ ^]^ which dynamically focus on the most relevant features, offer advantages for tabular data tasks where different features may be relevant for different instances.^[^ ^183^, ^184^, ^185^ ^]^ For example, TabNet^[^ ^183^ ^]^ uses attention‐based feature selection at each decision step, allowing it to dynamically prioritize important features per instance. This adaptability enhances model interpretability and helps capture non‐linear relationships across features, making attention‐based methods highly effective for complex tabular data.

Regularization techniques are particularly beneficial for tabular data, which often lacks structure and is prone to overfitting. Techniques such as Dropout,^[^ ^183^ ^]^ Batch Normalization,^[^ ^186^ ^]^ and L2 regularization help mitigate noise by constraining the learning process. For instance, Regularization Learning Network (RLN)^[^ ^187^ ^]^ applies trainable regularization coefficients to each weight in the neural network, reducing sensitivity and promoting sparsity. This learned regularization not only controls overfitting but also highlights the importance of remaining features, making RLN effective for tabular data tasks.

Multiplicative interaction modeling directly addresses the need to capture complex relationships between features in tabular data.^[^ ^188^, ^189^ ^]^ Models in this category explicitly construct interactions, either by pairing features multiplicatively or by learning logical functions. For example, DNF‐Net^[^ ^189^ ^]^ uses disjunctive normal form to capture multiplicative dependencies, effectively enabling the model to learn intricate feature combinations, which are often critical for tabular tasks.

Deep neural networks are effective in identifying complex, nonlinear relationships among diverse feature types, including numerical, textual, and graphical data. Recent research has applied these neural network strategies in material prediction, especially in zeolites,^[^ ^190^ ^]^ MOFs,^[^ ^191^ ^]^ and protein structure.^[^ ^192^ ^]^ The artificial neural network (ANN) techniques studied in CaO‐based sorbents^[^ ^193^ ^]^ displayed a good performance (R>0.99, MAPE< 0.34). However, these models generally require an extremely large volume of data to prevent overfitting, a requirement that tabular data often fails to meet. Furthermore, the application of deep learning in CaO‐based sorbent design presents additional challenges, such as the need for advanced methods to handle missing data, categorical features, and other pre‐processing tasks necessary to ensure model robustness and accuracy.

### 6.2 Tree‐Based Machine Learning

The early development of Gradient‐Boosted Decision Trees (GBDT)^[^ ^194^ ^]^ demonstrated exceptional performance on a wide range of tabular data tasks. GBDT operates by creating an ensemble of decision trees, where each tree is trained to correct the errors of its predecessors. By iteratively adding trees that minimize the remaining error, GBDT achieves high predictive accuracy and robustness. Building on GBDT, powerful variants such as XGBoost,^[^ ^194^ ^]^ LightGBM,^[^ ^195^ ^]^ and CatBoost^[^ ^196^ ^]^ have been developed. Among these, XGBoost is particularly versatile and widely used in industry^[^ ^197^, ^198^, ^199^ ^]^ due to its scalability, speed, and consistent performance across diverse applications and datasets.

Due to the typically limited and structured nature of data in materials science, regression models in general, and tree‐based models such as XGBoost in particular, perform well on small‐sample tabular datasets. This makes these models well‐suited for applications in materials design. XGboost^[^ ^194^ ^]^ regresses on the inputs and outputs for each stage, using a separate regressor for each output label, which shows high potential for application in the design on CaO‐based sorbents. XGBoost is particularly well‐suited to this task because, unlike deep learning models that generally require larger datasets, XGBoost has demonstrated strong performance with smaller data (<10K) sizes in previous studies.^[^ ^173^, ^200^ ^]^ Its enhancements in speed, scalability, and regularization further make it highly effective for capturing non‐linear interactions within tabular datasets, even when data is limited. Recent research^[^ ^198^ ^]^ displays a high prediction accuracy (R^2^ = 0.9699, MAPE = 0.0469, and MAE = 0.7683) of the XGBoost model in CaO‐based sorbents, which can effectively predict the capture efficiency of CO_2_ by CaO in a fluidized bed.

## 7 Conclusion

This paper explores recent advancements in CaO‐based sorbents within the realm of CaL technology, focusing on performance enhancements achieved through innovative synthesis methods and operational optimizations, by compiling a dataset of 1042 reported materials in order to identify broad patterns in performance. This investigation reveals that CaO‐based sorbents synthesized by sol‐gel, template, and combustion methods exhibit relatively large specific surface areas and enhanced stability. The introduction of dopants and chemical treatments with, e.g., citric acid, significantly enhance the structure of the CaO‐based sorbents, improving cyclic stability.

Furthermore, the incorporation of machine learning technologies provides novel approaches for the material design and process optimization. In particular, XGBoost demonstrates substantial potential in predicting the behavior of CaO‐based sorbents with smaller datasets (fewer than 10000 data points).

## Conflict of Interest

The authors declare no conflict of interest.

## Supporting information



Supporting Information
